# Determinants of under-nutrition among children under five years of age in Ethiopia

**DOI:** 10.1186/s12889-020-08539-2

**Published:** 2020-03-27

**Authors:** Gashu Workneh Kassie, Demeke Lakew Workie

**Affiliations:** 1 Department of Statistics, University of Gondar, P.O. Box: 96, Gondar, Ethiopia; 2 Department of Statistics, Bahir Dar University, P.O. Box: 79, Bahir Dar, Ethiopia

**Keywords:** Stunting, Underweight, Wasting, Partial proportional odds model, Single composite index, Ethiopia

## Abstract

**Background:**

Ethiopia is one of the developing countries where child under-nutrition is prevalent. Prior studies employed three anthropometric indicators for identifying factors of children’s under-nutrition. This study aimed at identifying the factors of child under-nutrition using a single composite index of anthropometric indicators.

**Methods:**

Data from Ethiopia’s Demographic and Health Survey 2016 was the base for studying under-nutrition in a sample of 9494 children below 59 months. A single composite index of under-nutrition was created from three anthropometric indices through principal component analysis recoded into an ordinal outcome. In line with World Health Organization 2006 Child Growth Standards, the three anthropometric indices involve z-score of height-for-age (stunting), weight-for-height (wasting) and weight-for-age (underweight). Partial proportional odds model was fitted and its relative performance compared with some other ordinal regression models to identify significant determinants of under-nutrition.

**Results:**

The single composite index of anthropometric indicators showed that 49.0% (19.8% moderately and 29.2% severely) of sampled children were undernourished. In the Brant-test of proportional odds model, the null hypothesis that the model parameters equal across categories was rejected. Compared to ordinal regression models, partial proportional odds model showed an improved fit. A child with mother’s body mass index less than 18.5 kg, from poorest family and a husband without education, and male to be in a severe under-nutrition status was 1.4, 1.8 1.2 and 1.2 times more likely to be in worse under-nutrition status compared to its reference group respectively.

**Conclusion:**

Authors conclude that the fitted partial proportional odds model indicated that age and sex of the child, maternal education, region, source of drinking water, number of under five children, mother’s body mass index and wealth index, anemic status of child, multiple births, fever of child before 2 months of the survey, mother’s age at first birth, and husband’s education were significantly associated with child under-nutrition. Thus, it is argued that interventions focus on improving household wealth index, food security, educating mothers and their spouses, improving maternal nutritional status, and increasing mothers’ health care access.

## Background

Malnutrition includes under-nutrition, and/or over nutrition. In developing countries, however, malnutrition is used to refer to under-nutrition [1]. The indicators of childhood under-nutrition involve stunting, wasting and underweight. Stunting, wasting and underweight occur in children below 59 months who are below 2 standard deviations from the median height-for-age, weight-for-height and weight-for-age, respectively as determined by the 2006 World Health Organization (WHO) Child Growth Standards [2].

Childhood under-nutrition is still prevalent in Sub-Saharan Africa. In this region, the prevalence of stunting, wasting and underweight for children under 5 years of age are 39, 10 and 25%, respectively [3]*.* Under-nutrition has both short- and long-term effects. Its short-term effects, for example, include mortality and morbidity. Global reports showed that around 45% of child deaths in developing countries is due to under-nutrition [4]. Its long-term effects include that children do not reach their full developmental potential and would have poor cognitive performance, which in turn has consequences on the country’s economic productivity [5]. The UNICEF conceptual framework depicts its general effects on childhood under-nutrition [6].

Prevalence of stunting and wasting or low weight-for-height decreased over the past decade though it remained high, with 38% of children under 5 years stunted and 10% wasted [7]. In 2000, for instance, 55.7% of Ethiopian children were stunted and this figure decreased to 43.4% in 2011, and to 40% in 2014 [8]. Child underweight prevalence was around 41% in 2000 and declined to 25% in 2014. Child wasting was also relatively higher than 10% in 2014 [9].

Several cross-sectional studies used the three anthropometric indicators separately to identify the factors associated to under-nutrition of children [9–13]. Nevertheless, owing to overlapping, none of these studies provided a comprehensive estimate of the undernourished children as stunning and wasting indicated chronic and acute under-nutrition respectively, while underweight is a composite indicator reflecting both acute and chronic under-nutrition, and it did not distinguish between anthropometric indicators [14–16]. Svedberg argues that to measure the overall prevalence of under-nutrition among young children sufficiently, a new aggregate indicator is needed, and proposed a composite index [14] as this kind of index measures the aggregate child under-nutrition derived from the conventional anthropometric indices (stunting, underweight and wasting) classifying them in to seven groups (no failure; stunted only; underweight only; wasted only; stunted and underweight; wasted and underweight; wasted, stunted, and underweight). Hence, a composite index is more useful over the conventional anthropometric indices for assessing the overall prevalence of under-nutrition and for identifying children with multiple anthropometric failures in a population. This study, therefore, tries to create a single composite index from the classical anthropometric indices and identify the risk factors associated with childhood under-nutrition in Ethiopia.

## Methodology

### Data source

2016 Ethiopia Demographic and Health Survey (EDHS) data was used for the analysis. The 2016 EDHS sample was stratified in to Urban and Rural areas and selected in two stages. In the first stage, a total of 645 enumeration areas (EAs) having an average of 181 households were selected with probability proportional to EA size (202 of them were from urban areas while 443 were rural). In the second stage, 28 households per EA were selected using systematic sampling. In all the selected households, height and weight measures were taken from 9494 children aged below 59 months [17]. Based on the household and women’s questionnaire, the children’s data including its complete anthropometric and valid age was analyzed. The household and women’s survey questionnaire are two questionnaires among five (the Household Questionnaire, the Woman’s Questionnaire, the Man’s Questionnaire, the Biomarker Questionnaire, and the Health Facility Questionnaire) that developed by Ethiopian Central Statistical Agency to reflect the population and health issues relevant to Ethiopia [17].

### Variables

#### Dependent variables

The three anthropometric variables are measured through z-scores of height-for-age, weight-for height and weight-for-age and are defined as: ${Z}_i=\frac{AI_i-\mu }{\sigma }$, where *AI*_i_ is the child anthropometric indicator, *μ* and *σ* refer respectively to median and standard deviation of the reference population.

The correlations between the three anthropometric variables are statistically significant (Table 1) and it is reasonable that a single composite index was created from HAZ, WHZ and WAZ using principal component analysis [18–20]. The first component alone explains 65.7% (Table 1) of the total variation of all anthropometric indices and this figure is significantly high enough to create a single composite index of under-nutrition [21]. Therefore, 0.81HAZ + 0.55WAZ + 0.15WHZ was taken as a new single composite index of under-nutrition which was again classified as nutrition status (severely undernourished if z-score < − 3, moderately undernourished if − 3 ≤ z-score < − 2.0 and nourished if z-score ≥ − 2.0). Then, the transformed variable was re-coded into ordinary outcome as ‘1’ = nourished, ‘2’ = moderately undernourished and ‘3’ = severely undernourished. The methodology for computing the indicators was based on the 2006 WHO Child Growth Standards [2].

**Table 1:** Eigen value, PC and correlation of the three anthropometric variables

Eigen value	Proportion	Cumulative	PC	HAZ (Corr)	WAZ (Corr)	WHZ (Corr)
4.026	0.657	0.657	1	0.81 (1)	0.55 (0.721)	0.15 (− 0.103)
2.090	0.341	0.997	2	0.40	−0.35	− 0.85 (0.602)
0.017	0.003	1.000	3	0.43	−0.75	0.51

Key: all correlations are statistically significant (*p* < 0.01); PC (principal Component); Corr (Correlation value); Values in each cell from column 5 to 7 are Principal component coefficients and correlations are in the parentheses

#### Explanatory variables

The selection of explanatory variables are theoretically driven that draw support from prior research with regard to factors affecting children’s nutritional status. Previous studies are referenced in creating categories for naturally continuous and discrete variables [22–26] (Table 2).

**Table 2:** Description and frequency of intermediate causes of child under-nutrition

Variables	Categories (Codes)	n (%)	Variables	Categories (Codes)	n (%)
Region	Tigray (1)	647 (6.8)	Mother’s Body mass index	Thin (0)	1840 (19.4)
	Afar (2)	90 (0.9)		Normal (1)	6974 (73.5)
	Amhara (3)	1865 (19.6)		Overweight (2)	585 (6.2)
	Oromia (4)	4164 (43.9)	Anemia level	Non Anemic (0)	6025 (63.5)
	Somali (5)	393 (4.1)		Anemic (1)	2948 (31.0)
	Benishangul (6)	100 (1.1)		Missing	521 (5.5)
	SNNPR (7)	1947 (20.5)	Had cough in last two weeks	No (0)	7571 (79.7)
	Gambela (8)	22 (0.2)		Yes (1)	1924 (20.3)
	Harari (9)	20 (0.2)	Had diarrhea recently	No (0)	8338 (87.8)
	Addis Adaba (10)	210 (2.2)		Yes (1)	1138 (12.0)
	Dire Dawa (11)	36 (0.4)		Missing	18 (0.2)
Type of place of residence	Urban (1)	1046 (11.0)	Had fever in last two weeks	No (0)	8087 (85.2)
	Rural (2)	8449 (89.0)		Yes (1)	1398 (14.7)
Sex of child	Male (1)	4851 (51.1)		Missing	10 (0.1)
	Female (2)	4644 (48.9)	Husband/partner’s education	No education (0)	4266 (44.9)
Age of child	0–5 (0)	1041 (11.0)		Primary (1)	3598 (37.9)
	6–11 (1)	1021 (10.8)		Secondary & above (2)	1086 (11.4)
	12–23 (2)	1896 (20.0)		Missing	544 (5.7)
	24–35 (3)	1788 (18.8)	House hold size	1–4 (small) (0)	2432 (25.6)
	36–47 (4)	1820 (19.2)		5–9 (medium (1)	6500 (68.5)
	48–59 (5)	1929 (20.3)		10 and more (Large) (2)	562 (5.9)
Birth order	1st (0)	1741 (18.3)	Source of drinking water	unimproved source (0)	4102 (43.2)
	2–3 (1)	2919 (30.7)		Improved source (1)	5278 (55.6)
	4–5 (2)	2276 (24.0)		Missing	115 (1.2)
	6 and more (3)	2558 (26.9)	Multiple birth	Single birth (0)	9270 (97.6)
Wealth index	Poorest (1)	2188 (23.0)		1st of multiple (1)	121 (1.3)
	Poorer (2)	2226 (23.4)		2nd of multiple (2)	104 (1.1)
	Middle (3)	2001 (21.1)	Educational level of mother	No education (0)	6225 (65.6)
	Richer (4)	1722 (18.1)		Primary (1)	2600 (27.4)
	Richest (5)	1358 (14.3)		Secondary & above (2)	670 (7.0)
No of children under five years in the household	1 (0)	5860 (61.7)	Age of mother at first birth	< 20 (0)	6139 (64.7)
	2 (1)	2651 (27.9)		20–34 (1)	3346 (35.2)
	3 or more (2)	983 (10.4)		35–49 (2)	10 (0.1)

### Statistics analysis

#### Ordinal logistic regression model

Logistic regression serves to model a categorical dependent variable as a function of one or more independent variables. The dependent variable may have two or more categories. When it has more than two categories, it may be ordered or unordered. Proportional Odds Model is instrumental to model the ordinal dependent variable through defining the cumulative probabilities rather than the probability of an individual event. The proportional odds model estimates the odds of being at or below a particular level of the response variable. It considers the probability of that event and all events before it. The proportional odds model is the default ordinal logistic regression type provided by statistical software [18, 19]. The proportional odds model with the logit or log-odds of the first *i* cumulative probabilities is modeled as a linear function of the explanatory variables as:

$$ Logit\;\left[{Y}_j\le i\left|{x}_j\right.\right]=\log \left[\frac{\pi_i\left({X}_j\right)}{1-{\pi}_i\left({X}_j\right)}\right]=\log\;it\left({\pi}_i\right)={\alpha}_i-{X}_j^{\prime}\beta, i=1,2,\dots 3C-1;j=1,2,\dots, n $$

If the proportional odds assumption is not met, then different models are needed to describe the relationship between each pair of outcome groups [21].

#### Generalized ordered logit model (GOLM)

The proportional odds assumption (**β** is independent of response level) may be too strict and needs testing. The GOLM adopts the parallel lines assumption for all C outcome categories. It permits the slope coefficients to vary for each of C-1 binary regressions [21]. The GOLM holds the nature of the proportional odds model (POM) by considering concurrently the effects of a collection of independent variables through successive dichotomizations of the outcome [27].

#### Partial proportional odds model (PPOM)

A partial proportional odds or non-proportional odds model relaxes the assumption of proportional odds. When the proportional odd assumption applies does not apply to all of the covariates, the partial proportional odds model may be used. This model allows some co-variables to be modeled with the proportional odds assumption, but for variables where the assumption is not satisfied, the effect associated with each *i*^*th*^ cumulative logit is increased by a coefficient (*γ*) adjusted by the other co-variables. The PPOM as articulated by Peterson and Harrell [28] imposes constraints for parallel lines only where they are needed. Thus, in this study the PPOM was employed which is defined as: $\log \left(\frac{pr\left(Y\le i\left|x\right.\right)}{pr\left(Y>i\left|x\right.\right)}\right)={\alpha}_i-\left(X{\beta}_i+{\tau \gamma}_i\right),i=1\dots C-1$, where **x** is vector containing the full set of independent variables, **τ** is a vector of subset independent variables that violate the parallel line assumption, and **γ**_**i**_ the regression coefficients associated with **τ**. The expected probabilities of belonging to a certain category are then defined by taking the exponential and rearranging the above equation in both sides.

#### Parameter estimation

Both the models defined above were fitted to the data set using STATA (version 14). The variable selection was purposeful and the first step is used to assess the parallel assumption. Firstly, a POM was fitted with command “ologit” and then the “Brant” test was performed to evaluate the parallel assumption. This test is used to compares the coefficients of beta from *C-1* binary logits and gives a list of variables violated the parallel assumption. The command “**gologit2**” was used to estimate the GOLM. The PPOM used to be estimated the usage of the **gologit2** command with the **autofit** choice to impose constraints on the variables where the parallel assumption was not violated [29].

All the ordinal logistic models are estimated via the technique of MLE. ML estimates are values of the parameters that have the ML of producing the observed sample. The likelihood equations lead to the unknown parameters in a non-linear function. The ordinal logistic regression model is fitted to the observed responses using the maximum likelihood approach. In general, the method of maximum likelihood produces values of the unknown parameters that best match the predicted and observed probability values.

#### Model selection

To compare the ordinal logistic models, the log-likelihood were calculated, and the model with a higher log-likelihood is taken as a better-fitting one. Models are compared based on the Akaike Information Criterion (AIC) and Baye’s Information Criterion (BIC) [30] and the models with the smaller absolute AIC and BIC statistic are preferred. The best model is selected from a set of competing models that has lowest value of AIC and BIC.

#### Test of overall model fit

The null hypothesis for an overall model fit test may be stated as “all the regression parameters are zero” while the alternative hypothesis is “at least one regression coefficient (parameter) is not zero”. To keep use of the selected mode, significance of the individual parameters is examined; the null hypothesis must be rejected. In addition, the goodness of fit of a model is tested by the deviance statistic [31].

## Results

### Data exploratory

Table 3 depicts the prevalence of under-nutrition of sampled children using each of the anthropometric measures separately and a single composite measure respectively. For instance, about 38.3% of sampled children were stunted (21.0% moderately and 17.3% severely). According to composite index of failure, out of sampled children in Ethiopia about 46.6% of children were undernourished. The single composite index of anthropometric indicators showed that 49.0% of sample children were undernourished (19.8% moderately and 29.2% severely).

**Table 3:** the prevalence of undernourished children by each anthropometric and a single composite measure

Anthropometric indicators	Nourished	Moderately undernourished	Severely undernourished
Stunting	5857 (61.7)	1990 (21.0)	1647 (17.3)
Wasting	8535 (89.9)	677 (7.1)	282 (3.0)
Underweight	7247 (76.3)	1616 (17.0)	631 (6.7)
New single Composite	4846 (51.0)	1880 (19.8)	2768 (29.2)

*N.B.* Numbers in each cell are frequencies and percentages are in the parentheses.

### Ordinal logistic regression models

As, the Brant test of parallel regression assumption violated (Chi-Square = 90.27, *p*-value = 0.000), proportional odds were excluded and generalized ordered logit model and partial proportional odds model were fitted to the data. Finally, a comparison of the models made (Table 4). The model which represents the best fit according to AIC and BIC is PPOM as it has the smallest AIC and BIC (Table 4) and it is also more parsimonious. Thus, PPOM was used to identify significant determinants of under-nutrition and parameter estimates of the PPOM are presented and interpreted for the significant predictors (at 5% significance level).

**Table 4:** Log-likelihood and likelihood ratio estimates

Model	Obs	LL(null)	LL (model)	DF	LR chi2	*P-*Value	AIC	BIC
GOLM	7910	− 8049.26	− 7269.13	84	1560.27	< 0.000	14,710.26	15,310.18
PPOM	7910	−8049.26	− 7283.19	54	1532.15	< 0.000	14,678.37	15,069.02

### Results of partial proportional odds model

Tables 5 and 6 show two result panels. The first (Table 5) contrasts the moderately and severely undernourished. In contrast to the remaining two categories of under-nutrition, signs of the coefficients in the first panel imply how likely nourishment of the child is.

**Table 5:** Maximum likelihood estimates of Partial proportional odds model

Predictors	Moderate and severe undernourished versus nourished					
	Coefficient	Std. Error	Z	P > |z|	Odds ratio	95% CI for OR
Region						
Afar	− 0.060	0.114	− 0.53	0.597	0.942	(0.754, 1.177)
Amhara	0.328	0.101	3.25	0.001	1.389	(1.140, 1.693)
Oromia	−0.372	0.096	−3.86	0.000	0.689	(0.571, 0.833)
Somali	−0.670	0.108	−6.21	0.000	0.512	(0.414, 0.632)
Benishangul	0.117	0.112	1.05	0.295	1.124	(0.903, 1.401)
SNNPR	−0.355	0.100	−3.54	0.000	0.701	(0.576, 0.853)
Gambela	−0.715	0.127	−5.65	0.000	0.489	(0.382, 0.627)
Harari	−0.213	0.127	−1.68	0.092	0.808	(0.630, 1.036)
Addis Ababa	−0.812	0.173	−4.69	0.000	0.444	(0.316, 0.624)
Dire Dawa	−0.097	0.138	−0.70	0.485	0.908	(0.692, 1.191)
Residence						
Rural	−0.138	0.104	−1.32	0.186	0.871	(0.711, 1.069)
Mother’s education						
Primary	−0.100	0.061	−1.62	0.105	0.905	(0.803, 1.021)
Secondary &>	−0.547	0.116	−4.72	0.000	0.579	(0.461, 0.726)
Drinking water						
Unimproved	0.102	0.051	2.01	0.045	1.108	(1.002, 1.224)
House hold size						
5–9	0.054	0.069	0.78	0.434	1.055	(0.922, 1.207)
10 and more	0.208	0.123	1.70	0.089	1.232	(0.969, 1.567)
No children < 5 years						
2	−0.144	0.076	−1.90	0.057	0.866	(0.746, 1.004)
3 and more	0.150	0.055	2.72	0.007	1.162	(1.043, 1.294)
Wealth index						
Poorer	0.082	0.070	1.18	0.238	1.085	(0.947, 1.244)
Middle	−0.204	0.076	−2.67	0.008	0.815	(0.702, 0.947)
Richer	−0.453	0.083	−5.44	0.000	0.636	(0.540, 0.748)
Richest	−0.447	0.113	−3.96	0.000	0.639	(0.512, 0.798)
Anemia						
Anemic	0.197	0.050	3.95	0.000	1.218	(1.105, 1.344)
Husband’s Education						
Primary	− 0.087	0.059	−1.47	0.141	0.916	(0.816, 1.030)
Secondary &>	−0.171	0.086	−2.00	0.045	0.924	(0.769, 1.109)
Birth order						
2–3	−0.059	0.074	−0.79	0.428	0.943	(0.816, 1.090)
4–5	−0.035	0.091	−0.38	0.701	0.966	(0.809, 1.154)
6 and more	−0.079	0.093	−0.85	0.395	0.924	(0.769, 1.109)
Multiple birth						
1st of multiple	0.725	0.210	3.45	0.001	2.066	(1.369, 3.117)
2nd of multiple	0.796	0.222	3.58	0.000	2.216	(1.430, 3.427)
Sex						
Female	−0.110	0.049	−2.26	0.024	0.895	(0.814, 0.985)
Age of child in month						
6–11	0.583	0.123	4.74	0.000	1.792	(1.408, 2.281)
12–23	1.646	0.107	15.37	0.000	5.185	(4.203, 6.395)
24–35	2.057	0.107	19.19	0.000	7.819	(6.338, 9.647)
36–47	1.980	0.107	18.44	0.000	7.244	(5.869, 8.941)
48–59	1.859	0.107	17.33	0.000	6.415	(5.199, 7.916)
Had fever						
Yes	0.237	0.077	3.06	0.002	1.267	(1.089, 1.475)
Had cough						
Yes	−0.019	0.074	−0.26	0.793	0.981	(0.849, 1.133)
Mother’s age at 1st birth						
20–34	−0.110	0.049	−2.24	0.025	0.895	(0.813, 0.986)
35–49	0.296	0.562	0.53	0.599	1.344	(0.447, 4.046)
BMI of mother						
Normal	−0.349	0.055	−6.36	0.000	0.705	(0.633, 0.785)
Overweight	−0.939	0.106	−8.87	0.000	0.391	(0.318, 0.481)
Constant	−0.805	0.189	−4.26	0.000	0.447	(0.309, 0.647)

**Table 6:** Maximum likelihood estimates of Partial proportional odds model

Predictors		Severely undernourished versus nourished and moderately undernourished					
		Coefficient	Std. Error	Z	P > |z|	Odds ratio	95% CI for OR
Region	Afar	0.198	0.115	1.73	0.084	1.219	(0.974, 1.525)
	Amhara	0.328	0.101	3.25	0.001	1.389	(1.139, 1.693)
	Oromia	−0.152	0.102	−1.49	0.136	0.859	(0.704, 1.049)
	Somali	−0.312	0.113	−2.76	0.006	0.732	(0.586, 0.914)
	Benshangul	0.293	0.114	2.57	0.010	1.341	(1.073, 1.677)
	SNNPR	−0.007	0.105	− 0.07	0.946	0.993	(0.808, 1.220)
	Gambela	−0.439	0.141	−3.12	0.002	0.645	(0.489, 0.849)
	Harari	−0.213	0.127	−1.68	0.092	0.808	(0.630, 1.036)
	Addis Ababa	−0.812	0.173	−4.69	0.000	0.444	(0.316, 0.624)
	Dire Dawa	0.237	0.143	1.66	0.097	1.267	(0.958, 1.676)
Residence	Rural	−0.138	0.104	−1.32	0.186	0.871	(0.711, 1.069)
Mother’ education	Primary	−0.100	0.061	−1.62	0.105	0.905	(0.803, 1.021)
	Secondary & above	−0.547	0.116	−4.72	0.000	0.579	(0.461, 0.726)
Drinking water	Unimproved	0.102	0.051	2.01	0.045	1.108	(1.002, 1.224)
House hold size	5–9	0.054	0.069	0.78	0.434	1.055	(0.922, 1.207)
	10 & more	0.208	0.123	1.70	0.089	1.232	(0.969, 1.566)
No children< 5 years	2	−0.144	0.076	−1.90	0.057	0.866	(0.746, 1.004)
	3 and more	0.150	0.055	2.72	0.007	1.162	(1.043, 1.294)
Wealth index	Poorer	0.082	0.070	1.18	0.238	1.085	(0.947, 1.244)
	Middle	−0.204	0.076	−2.67	0.008	0.815	(0.702, 0.947)
	Richer	−0.453	0.083	−5.44	0.000	0.636	(0.540, 0.748)
	Richest	−0.612	0.121	−5.06	0.000	0.542	(0.428, 0.688)
Anemia	Anemic	0.197	0.050	3.95	0.000	1.218	(1.105, 1.343)
Husband’s education	Primary	−0.188	0.063	−3.01	0.003	0.828	(0.732, 0.936)
	Secondary & above	−0.171	0.086	−2.00	0.045	0.843	(0.712, 0.996)
Birth order	2–3	−0.059	0.074	−0.79	0.428	0.943	(0.816, 1.090)
	4–5	0.096	0.093	1.03	0.302	1.101	(0.917, 1.320)
	6 and more	0.050	0.095	0.53	0.598	1.052	(0.872, 1.268)
Multiple birth	1st of multiple	0.725	0.210	3.45	0.001	2.066	(1.369, 3.112)
	2nd of multiple	0.796	0.222	3.58	0.000	2.216	(1.433, 3.427)
Sex	Female	−0.214	0.053	−4.07	0.000	0.808	(0.729, 0.895)
Age of child in month	6–11	0.583	0.123	4.74	0.000	1.792	(1.408, 2.281)
	12–23	1.646	0.107	15.37	0.000	5.185	(4.203, 6.395)
	24–35	2.057	0.107	19.19	0.000	7.819	(6.338, 9.647)
	36–34	1.980	0.107	0.000	0.000	7.244	(5.869, 8.941)
	48–59	1.859	0.107	17.33	0.000	6.415	(5.199, 7.916)
Fever	Yes	0.237	0.077	3.06	0.002	1.267	(1.089, 1.475)
Cough	Yes	−0.019	0.074	−0.26	0.793	0.981	(0.849, 1.133)
Mother’s age at birth	20–34	−0.110	0.049	−2.24	0.025	0.895	(0.813, 0.986)
	35–49	0.296	0.562	0.53	0.599	1.344	(0.447, 4.046)
BMI of mother	Normal	−0.349	0.055	−6.36	0.000	0.705	(0.633, 0.785)
	Overweight	−0.939	0.106	−8.87	0.000	0.391	(0.318, 0.481)
Constant		−1.929	0.191	10.09	0.000	0.145	(0.100, 0.211)

**Key:** the reference category for predictors is: Region (Tigray), Residence (urban), mother’s education (no education), source of drinking water (improved source), house hold size (1–4), number of children < 5 years (1), wealth index (poorest), anemia (no), husband’s education (no education), birth order (1), multiple birth (single), sex (male), age of child (0–6), diarrhea (no), cough (no), fever (no), mother’s age at birth (< 20), BMI (thin); BMI (Body mass index); Std. Error (Standard error); CI (confidence Interval) OR (Odds Ratio).

Similarly, the second panel (Table 6) contrasts the severely undernourished category with nourished and moderately undernourished.

Hence, positive coefficients indicate that higher category values on the predictor make it more likely that the respondent will be in a higher category than the current one, while negative coefficients indicate that higher category values on the predictor increase the likelihood of being in the current or a lower category.

From partial proportional odds model the categories Afar, Oromia, Somali, SNNPR, Gambela, Harari, Dire Dawa; as well as richest wealth index; husband’s education, birth order and sex violated the parallel lines assumption. The model therefore allows the coefficients of these variables to vary across the two equations. From PPOM results, region, mother’s education and source of drinking water, number of under five children, wealth, anemia, multiple birth, age and sex of the child, had fever, mother’s age at first birth and body-mass index, and husband’s educational level were significantly related with under-nutrition.

### Predictors that do not violate the parallel line assumption

The results of PPOM revealed that holding all variables constant, as compared to a child in Tigray, a child in Amhara was 1.4 (OR = 1.4; CI: 1.14–1.69) times more likely to be in moderate or severe under-nutrition status. Similarly, compared to a child in Tigray, a child in Amhara was 1.4 (*p*-value =0.0001) times more likely to be in severe rather than in the moderate or nourished under-nutrition statuses. Holding other variables constant, the odds of being undernourished was worse, 2.3(OR = 0.44; CI: 0.32–0.62) times in children of Tigray’s as compared to children of Addis Ababa.

The fitted model showed that compared with children whose mother had secondary or higher education, children from uneducated mother have risk of an even worse under-nutrition status 1.8 = (OR = 0.58; CI: 0.46–0.73) compared with the children with illiterate fathers, children with secondary or higher educated fathers were around 8% (OR = 0.92; *p*-value =0.045) and less likely to be in the worst under-nutrition status. The risk of being in an even worse under-nutrition status decreased by 11% (OR = 0.89; *p*-value =0.025) in a child born from a mother aged 20–34 years as compared to a child born from a mother aged < 20 years. Keeping all other variables constant, as opposed to a child who had normal and obese mother, a child with mother of BMI less than18.5 kg was 1.4 (OR = 0.71; CI: 0.63–0 .79) and 2.6 (OR = 0.39; CI: 0.31–0.65) times more likely to be in worse under-nutrition status respectively.

The results of this study revealed that as compared to children from families having a single child aged under 5 years, the odds of being in a worse under-nutrition status were 1.2 (OR = 1.2; *p*-value =0.007) times higher for children from families having 3 or more under-five children. Children from households with poorest wealth index, the risk of undernourishment decreased by 18% (OR = 0.82; *p*-value =0.008) and by 36% (OR = 0.64; *p*-value = 0.000) in children from families with middle wealth and richer wealth index respectively. The first and second born of the multiple children were 2.1 (OR = 2.1; *p*-value =0.001) and 2.2 (OR = 2.2; CI: 1.43–3.43) times more likely to have a respectively worse under-nutrition status as compared to children from a family of single birth. Holding all other variables constant, children aged 6–11, 12–23, 24–35, 36–47 and 48–59 months were respectively 1.8 (OR = 1.8; CI: 1.4–2.3), 5.2 (CI: 4.2–6.4), 7.8 (CI: 6.3–9.6), 7.2 (CI: 5.9–8.9) and 6.4 (CI: 5.2–7.9) times more likely to be in a worse under-nutrition status as opposed to children aged 0–6 months.

Holding all variables constant, the fitted model indicated that the risk of having worse under-nutrition status was 1.2 (OR = 1.2; CI: 1.1–1.3) times higher among anemic children when compared to the non-anemic children. Compared to children who had no fever, the risk of being in a worse under-nutrition status was 1.3 (OR = 1.3; *p*-value = 0.002) times higher among children who had fever in the last 2 weeks before the survey. Compared to children from household who have consumed water from improved source, the odds of being undernourished increased by 10% (OR = 1.1; *p*-value = 0.045) among children from households who have not consumed water from improved source.

### Predictors that violate the parallel regression assumption

The results of PPOM showed that compared to a child from Oromia, Somali, SNNP and Gambella, a child from Tigray region was 1.4 (OR = 0.69; CI: 0.58–0.83), 2 (OR = 0.50; CI: 0.41–0.63), 1.4 (OR = 0.70; CI: 0.58–0.85) and 2 (OR = 0.49; CI: 0.38–0.63) times more likely to be in a moderate or severe under-nutrition. Compared to a child in Somali and Gambella, a child from Tigray region was 1.4 (*p*-value =0.006) and 1.5 (*p*-value =0.002) times more likely to be in a severe rather than in a nourished or moderate under-nutrition status. In Contrast to a child from Tigray region, a child from Benshangul region was 1.34 (*p*-value =0.01) times more likely to be in a severe under-nutrition status rather than in a nourished or moderate under-nutrition status. The children from families with poorest wealth index were found 1.5 (OR = 0.64; CI: 0.51–0.80) times more likely to be in moderate or severe under-nutrition rather than in a nourished status when compared to children with richest wealth index households. Keeping all other variables constant, in contrast to children from families with richest wealth index, the children with families of poorest wealth index were 1.8 (OR = 0.54; CI: 0.43–0.69) times more likely to be in a severe rather than nourished or moderate under-nutrition status. In comparison to females, the fitted model had showed that male children were 1.1 (*p*-value =0.024) times more likely to be in moderate or severe under-nutrition rather than in a nourished status. In contrast to female children, the risk of male children to be in a severe under-nutrition status was 1.2 (OR = 0.81; CI: 0.73–0.89) times higher than those children in a nourished or moderate status as compared to children born to husband with secondary or higher education. Holding all other variables constant, the risk of children born from a husband without education to be in severe under-nutrition status was 1.2 (*p*-value =0.003) times higher than those in a nourished or moderate status.

## Discussion

In this study, a single composite index of under-nutrition was computed based on principal component analysis technique from the three classical anthropometric indices and recoded into ordinal outcome. Generalized ordered logit model and partial proportional odds model were fitted to the data and comparisons of models made. Thus, the best fit according to AIC and BIC is PPOM and it was used to identify significant determinants of under-nutrition and parameter estimates of the PPOM are presented and interpreted for the significant predictors (at 5% significance level). Significant factors that associated with child under-nutrition consist of the child’s region, sex and age; mother’s age at first birth, mother’s and husband’s education, mother’s body mass and wealth index, multiple births, number of children under five, source of drinking water, children fever before 2 months and level of anemia.

This study found that age of the child significantly affected the child’s nutritional status. This finding is consistent with findings from studies in Ethiopia and Bangladesh [23, 26, 32, 33] which found out that risk of undernourishment increased along with increase in the age of a child. This is attributable to late introduction of supplementary food with low nutritional quality [34]. The study further indicated that among Ethiopian under-five children, males rather than females were more likely to be underweight, stunted or wasted. This result complies with findings from previous studies from Ethiopia and Burkina Faso [13, 23, 35]. A possible explanation is that childhood morbidity is higher among males rather than among females, even after adjusting for gestational age and body size [35]. This finding was contrasted with finding of previous studies [32, 36] which reported that females were more prone to under-nutrition. However an Ethiopian study reported that while male children more susceptible to stunting, female children had more susceptibility to under-weight-ness [37].

This study identified maternal education as potential determinant factor of under-nutrition. Compared with a child with higher maternal education, a child with lower maternal education has more risk of being in a worse under-nutrition. Several previous studies from Ethiopia, Bangladesh, Tanzania and Uganda complied with this finding supporting the notion that children whose mothers have higher education were less likely to be undernourished as compared with children whose mother had lower or no education [22, 26, 33–35, 38–41] indicating that maternal education is an essential factor affecting infant feeding practices. As educated mothers have better knowledge of child health and nutrition, they are more conscious of their child’s health and look after their children better [42]. Not only maternal education but also father’s education significantly associated with childhood under-nutrition, and children whose father attended formal education had less chance of being undernourished. Other studies reported similar findings [35]. Fathers with formal education know better about proper child feeding and hygiene practices, which contribute positively to preventing childhood under-nutrition [43].

This study found a significant association between maternal BMI and child’s nutritional status. Compared to a child born from a normal or obese mother, a child from a thin mother is more likely to have a worse under-nutrition status. In contrast to mothers with normal BMI, mothers who had a lower BMI marginally associated with higher odds of childhood under-nutrition. Prior studies support results of this study [22, 23, 33, 35, 39, 40, 44]. As Maternal BMI is an important determinant of child under-nutrition and is influenced by maternal nutrition, in order to improve child growth, proper nutrition is essential for the mothers during the prenatal and postnatal period. Healthier mothers have less risk of having undernourished children [39]. In this study, mother’s age at first birth was identified as a determinant factor of under-nutrition. Findings from similar studies in Democratic Republic of Congo revealed that, compared to the children with mothers below 20 years of age at childbirth, children with the mothers aged 20–34 years had significantly lower odds of being stunted [24].

Similar to findings from previous studies in Ethiopia [26, 32], this study also found that region is a significant factor of under-nutrition. A child living in Tigray has a higher risk of under-nutrition compared to a child in Addis Ababa, Oromia, Somali, SNNP, and Gambella, while compared to a child in Amhara and Benshangul, it has lower risk of under-nutrition and this finding is consistent with findings from other studies in Ethiopia [26]. The study further indicated that wealth index significantly associated with nutritional status. Accordingly, a child from a lower category of wealth index household has a higher risk of being undernourished, which complies with findings from previous studies in different developing countries [23, 26, 33–35, 39]. This could be explained in that increased income improves dietary diversity [45], which in turn improves the adequacy of nutrient intake and nutritional status.

Findings of this study showed that the higher the number of children under 5 years in the household, the higher the risk the child will be undernourished. Findings from prior studies in Ethiopia [23, 25] identified that number of under-five children per household as a significant determinant in aggravating under-nutrition. This study found that compared to the non-anemic children, the risk under-nutrition was higher among anemic children, a conclusion which complies with findings from previous studies [35, 46]. Compared to children who had no fever 2 weeks before the survey, the risk of under-nutrition increased for children who had fever 2 weeks prior to the survey, a finding which complies with previous studies in Ethiopia and Burkina Faso [23, 25]. Consistent with results from studies of [23, 46], compared to a singleton child at birth, children from mothers who had multiple births were more likely to have a worse under-nutrition. Finally, source of drinking water was a significant factor of under-nutrition in that compared to children who have an improved water source, children from a families without an improved source of water were more likely to have a worse under-nutrition, which aligns with findings from previous studies by Poda et al in Burkina Faso, Zeray et al in Ethiopia and Tasnim in Developing Countries [23, 25, 41].

## Conclusion

This study aimed to identify the risk factors associated with childhood under-nutrition in Ethiopia using a single composite index as response variable created from the traditional anthropometric indices (stunting, wasting and underweight). The single composite index of anthropometric indicators showed that 49.0% (19.8% moderately and 29.2% severely) of sampled children were undernourished.

The Brant test of proportional odds model indicated the null hypothesis that the model parameters are equal across categories was rejected. Based on AIC, partial proportional odds model indicated an improved fit compared to ordinal regression models. Hence, the fitted partial proportional odds model revealed child’s age, maternal education, region, source of drinking water, number of children under 5 years, wealth index, anemic status of child, multiple birth, child’s sex, fever, mother’s age at birth, body mass index of mother and husband’s education were significantly associated with children under-nutrition.

It is recommended that nutritional status of children be improved though multi-factorial interventions. Policy measures that reduce burden of under-nutrition can be implemented to increase access to health care through providing essential services. Further research may explore the specific factors responsible for gender and region disparities in the prevalence of under-nutrition among children, and assess the spatial epidemiology of child under-nutrition in Ethiopia in order to identify the hotspots of child under-nutrition.

## Data Availability

The data set supporting the conclusions of this article is held by the authors and the Central Statistical Agency, CSA, Ethiopia, and the de-recognized data may be made available if a unique request is crafted from CSA website (https://www.csa.gov.et).

## Abbreviations

AIC: Akaike Information Criteria
BIC: Bayesian information criteria
BMI: Body mass index
CI: Confidence interval
CSA: Central statistical agency
EAs: Enumeration areas
EDHS: Ethiopia demographic and health survey
GOLM: Generalized ordered logit model
HAZ: Height for age z-score
LL: Log likelihood
Logit: Log of odds
ML: Maximum likelihood
MLE: Maximum likelihood estimation
OLS: Ordinary least square
OR: Odds ratio
PCA: Principal component analysis
POM: Proportional odds model
PPOM: Partial proportional odds model
UN: United nation
UNICEF: United Nations Children’s Fund
WAZ: Weight for age z-score
WHO: World Health Organization
WHZ: Weight for height z-score
